# C5a Regulates IL-1β Production and Leukocyte Recruitment in a Murine Model of Monosodium Urate Crystal-Induced Peritonitis

**DOI:** 10.3389/fphar.2017.00010

**Published:** 2017-01-23

**Authors:** Hanif J. Khameneh, Adrian W. S. Ho, Federica Laudisi, Heidi Derks, Matheswaran Kandasamy, Baalasubramanian Sivasankar, Gim Gee Teng, Alessandra Mortellaro

**Affiliations:** ^1^Singapore Immunology Network (SIgN), Agency for Science, Technology and Research (A*STAR)Singapore, Singapore; ^2^Singapore Institute for Clinical Sciences (SICS), Agency for Science, Technology and Research (A*STAR)Singapore, Singapore; ^3^Division of Rheumatology, University Medicine Cluster, National University Health System (NUHS)Singapore, Singapore; ^4^Department of Medicine, Yong Loo Lin School of Medicine, National University of Singapore (NUS) and National University Health System (NUHS)Singapore, Singapore

**Keywords:** C5a, gout, IL-1β, neutrophils, NLRP3 inflammasome

## Abstract

Gouty arthritis results from the generation of monosodium urate (MSU) crystals within joints. These MSU crystals elicit acute inflammation characterized by massive infiltration of neutrophils and monocytes that are mobilized by the pro-inflammatory cytokine IL-1β. MSU crystals also activate the complement system, which regulates the inflammatory response; however, it is unclear whether or how MSU-mediated complement activation is linked to IL-1β release *in vivo*, and the various roles that might be played by individual components of the complement cascade. Here we show that exposure to MSU crystals *in vivo* triggers the complement cascade, leading to the generation of the biologically active complement proteins C3a and C5a. C5a, but not C3a, potentiated IL-1β and IL-1α release from LPS–primed MSU-exposed peritoneal macrophages and human monocytic cells *in vitro*; while *in vivo* MSU–induced C5a mediated murine neutrophil recruitment as well as IL-1β production at the site of inflammation. These effects were significantly ameliorated by treatment of mice with a C5a receptor antagonist. Mechanistic studies revealed that C5a most likely increased NLRP3 inflammasome activation via production of reactive oxygen species (ROS), and not through increased transcription of inflammasome components. Therefore we conclude that C5a generated upon MSU-induced complement activation increases neutrophil recruitment *in vivo* by promoting IL-1 production via the generation of ROS, which activate the NLRP3 inflammasome. Identification of the C5a receptor as a key determinant of IL-1-mediated recruitment of inflammatory cells provides a novel potential target for therapeutic intervention to mitigate gouty arthritis.

## Introduction

Gout is the most common form of inflammatory arthritis and is associated with a substantial public health burden (Rees et al., 2014). The clinical prognosis for gout remains unsatisfactory, considering the intense pain of acute flares, loss of productivity, impairment of physical function, and long-term complications, including morbidities and mortality (Rees et al., 2014). Novel therapeutic strategies for gout are therefore needed, especially for patients who are refractory to or intolerant of conventional treatment (e.g., non-steroidal anti-inflammatory drugs, colchicine, or glucocorticoids). Emerging data have shown that aberrant expression of complex signaling pathways are involved in the inflammation of gout (Santegoets et al., 2011; Jin et al., 2014). Understanding the specific mechanisms driving the production of key cytokines involved in these pathways may offer improved prospects for the development of more efficacious and less toxic therapies (Dinarello and van der Meer, 2013; Goh et al., 2014; Perez-Ruiz et al., 2014).

Gout is caused by deposition of monosodium urate (MSU) crystals into the joints, which provokes a massive infiltration of inflammatory cells, mainly neutrophils and monocytes (Dalbeth et al., 2016). The interaction of these immune cells with the MSU crystals heightens inflammation, leading to further recruitment of increasing numbers of immune cells into the joint and escalation of the condition. IL-1 plays a pivotal role in gout-associated inflammation by instigating the release of multiple pro-inflammatory cytokines and chemokines (IL-8, IL-6, CXCL8, CXCL1), and the upregulation of adhesion molecules (selectins, integrins) on endothelial cells, which directly induces a massive infiltration of inflammatory cells (i.e., neutrophils and monocytes) at the site of crystal deposition (Brown et al., 1994; Hachicha et al., 1995). Prolonged accumulation of these cells can result in irreversible destruction of joint tissues and increases the risk of chronic inflammation. Therapies directed toward mitigating MSU-induced neutrophil infiltration and activation may therefore be beneficial for gout patients.

The exact mechanisms driving the release of IL-1β had remained elusive until the discovery that MSU crystals activate the NLRP3 inflammasome in myeloid cells of the immune system (Martinon et al., 2006; Giamarellos-Bourboulis et al., 2009; Kingsbury et al., 2011). NLRP3 belongs to the family of intracellular NOD-like receptors that, upon sensing of pathogen- or danger-associated molecular patterns (PAMPs and DAMPs, respectively), recruits the adaptor protein ASC, which aggregates and forms the inflammasome complex (Zambetti et al., 2012; Broderick et al., 2015). ASC molecules, in turn, recruit the precursor of caspase-1 (pro-casp-1), which undergoes autoproteolysis and is then able to process pro-IL-1β and pro-IL-18 to their active forms (Zambetti et al., 2012; Broderick et al., 2015).

*In vitro*, MSU crystals alone, much like other particulates (e.g., calcium pyrophosphate dihydrate crystals, silica, aluminum hydroxide), are not able to activate the NLRP3 inflammasome autonomously unless a “priming” pattern recognition signal, such as TLR ligands or proinflammatory cytokines, is provided alongside (Bauernfeind et al., 2009; Franchi et al., 2009): this priming signal is required for the transcription of pro-IL-1β and NLRP3 genes, whose products are activated by the second stimulus. However, *in vivo*, inflammatory responses are elicited by MSU crystals in the sterile environment of the joint space, implying that myeloid cells in this context have already received a priming signal, the source of which is currently unknown.

One of the mechanisms through which MSU crystals can induce inflammation is through activation of the complement system (Giclas et al., 1979). The complement cascade can be activated by the classical, lectin, and alternative pathways, with all pathways converging at the formation of the C3 and C5 convertases, which are required for the generation of the biologically active complement peptides (also known as anaphylatoxins), C3a and C5a, as well as the formation of the membrane attack complex (MAC) on target cells (Merle et al., 2015a). MSU crystals can activate both the classical and alternative pathways of the complement system (Doherty et al., 1983). In particular, formation of an active C5 convertase on the crystal surface was shown to trigger the cleavage of C5 proteins into C5a and C5b subunits (Russell et al., 1982). C5a is a potent chemoattractant factor for neutrophils and monocytes (Ricklin et al., 2010), and can also elicit the production of inflammatory cytokines, including IL-1β, as well as chemokines released by both endothelial cells and phagocytes (i.e., macrophages and dendritic cells; Laudes et al., 2002; Monsinjon et al., 2003).

Activated complement components have been detected in synovial fluids from gout patients during acute attacks (Doherty et al., 1988; Brodeur et al., 1991). Moreover, the MAC has been shown to promote neutrophil recruitment in a rabbit model of experimental gout arthritis (Tramontini et al., 2004). However, the chemoattractant properties of C5a and C3a have yet to be well-investigated in the context of gouty inflammation: indeed, it remains unclear whether or how these anaphylatoxins might instigate neutrophil and monocyte recruitment during crystal deposition, or contribute to IL-1β release *in vivo*.

In this study, we show that MSU crystals activate the complement system in myeloid cells, leading to generation of C3a and C5a. C5a, but not C3a, regulates IL-1β release *in vitro* and *in vivo*, and is thereby responsible for the recruitment of neutrophils and monocytes to the site of MSU-induced inflammation. Blockade of C5a receptor signaling markedly decreased leukocyte infiltration, and suggests that therapeutic targeting of C5a may prove beneficial in treating acute and chronic gouty arthritis.

## Materials and Methods

### Mice

C57BL/6 (B6) and Balb/c mice were purchased from the Biological Resource Center ((BRC), Agency for Science, Technology, and Research (A^∗^STAR), Singapore). C3^-/-^ (stock # 003641; on B6 background), C5ar1^-/-^ (stock #006845) and C3ar1^-/-^ mice (stock # 005712; both on Balb/c background) were from The Jackson Laboratory. Double C3ar1^-/-^ and C5ar1^-/-^ mice were generated by crossing C5ar1^-/-^ and C3ar1^-/-^ mice. All experiments were conducted with gender- and age-matched mice, and all mutants were backcrossed to B6 or Balb/c backgrounds for at least 10 generations. Animals were bred under specific pathogen-free conditions at the BRC (A^∗^STAR, Singapore) and experiments were performed under the approval of the Institutional Animal Care and Use Committee (IACUC) in compliance with the Law and Guidelines for Animal Experiments of the BRC (A^∗^STAR, Singapore).

### *In vitro* Stimulation of Peritoneal Macrophages

Mice received an intraperitoneal (i.p.) injection of Brewer thioglycollate medium (3%) to increase macrophage yield and euthanized 3 days later. Peritoneal macrophages were collected by lavage of the peritoneal cavity with 10 ml fresh RPMI medium. Red blood cell lysis was performed using hypotonic ammonium chloride solution (0.084%) for 5 min at room temperature. Peritoneal macrophages were plated at 2 × 10^5^/well in 96-well plates, and stimulated with *E. coli* LPS (0.1 μg/ml) for 3 h with or without MSU (250 μg/ml) for an additional 4 h, in the presence of recombinant C3a (0.25 and 1.25 μg/ml) or C5a (1–10–50 ng/ml). After 24 h, cell culture supernatants were collected.

Human monocytic (THP1) cells were plated at 1 × 10^5^/well in 96-well plates and treated with PMA (200 nM) overnight to differentiate monocytes into macrophages. Cells were then washed and exposed to *E. coli* LPS (0.1 μg/ml) for 3 h with or without MSU (250 μg/ml) for an additional 4 h, in the presence of recombinant C5a (1–10–50 ng/ml). After 24 h, cell culture supernatants were collected.

### Monosodium Urate (MSU)-Mediated Peritonitis

Eight- to ten-week-old wild-type (WT), C3^-/-^, C3ar^-/-^, C5a^-/-^, and C3ar^-/-^ C5ar^-/-^ mice were injected i.p. with 3 mg MSU crystals in 0.5 ml of saline. Control mice were injected with saline alone. After 6 h, mice were euthanized with CO_2_ and peritoneal exudate cells were collected by lavage with cold medium. The resulting cell suspensions were centrifuged before red blood cell lysis using hypotonic ammonium chloride solution for 5 min at room temperature, then quantification of cell number. In some experiments, mice received linear C5a receptor antagonistic peptide (1 mg/kg of mouse weight, AnaSpec Inc) by i.p. injection 5 min prior to MSU treatment.

### Flow Cytometry

Total peritoneal cells were labeled with CD11b-APC (M1/70, eBioscience), Ly6C-PE-Cy7 (HK1.4, Biolegend), and Ly6G-PE (1A8, BD PharMingen) for 15 min at room temperature. Cells were then washed with PBS twice before analysis using a Fortessa flow cytometer (BD Biosciences). Data were analyzed using FlowJo (Treestar). Neutrophils were identified as CD11b^+^ Ly6G^+^ cells and monocytes as CD11b^+^ Ly6C^+^ Ly6G^-^ cells.

### ELISA

IL-1α and IL-1β were measured using DuoSet ELISA kits (R&D Systems), following the manufacturer’s instructions. C3a and C5a levels in peritoneal lavage fluids were quantified by a sandwich ELISA as previously described (Kandasamy et al., 2013). The following antibody pairs were used: rat anti-mouse C3a or C5a capture antibody (2 μg/ml, overnight, 4°C), and biotinylated rat-anti mouse C3a or C5a detection antibody (2 μg/ml, 1 h, room temperature), all from BD Pharmingen. The absorbance was read at 450 nm with the reference wavelength at 570 nm, using a Tecan M200 Infinite plate reader (Tecan).

### Quantitative RT-PCR

Quantitative RT-PCR was performed using the following validated SYBR Green primers: Nlrp3, 5′-CCTCTAGCTTCTGCCGTGGTCTCT-3′ and 5′-CGAAGCAGCATTGATGGGACA-3′; Asc, 5′-CTGAGCAGCTGCAAACGACTAAA-3′ and 5′-CTTCTGTGACCCTGGCAATGAGT-3′; Casp1, 5′-CTGTCAGGGGCTCACTTTTCATTG-3′ and 5′-AATGTCCCGGGAAGAGGTAGAAAC-3′; Il1a, 5′-TCAGCACCACTTGGTAAATGAC-3′ and 5′-GTGTTTCTGGCAACTCCTTCAGC-3′; Il1b, 5′-GGTCAAAGGTTTGGAAGCAG-3′ and 5′-TGTGAAATGCCACCTTTTGA-3′; Gapdh, 5′-TCGTCCCGTAGACAAAATGG-3′ and 5′-TTGAGGTCAATGAAGGGGTC-3′. Amplification was performed using an Applied Biosystems 7500 Real-Time PCR System. The relative expression level of each gene was evaluated using the ΔΔC_t_ method. The difference between the C_t_ of the target gene and the C_t_ of the Gapdh housekeeping gene was normalized to the ΔC_t_ of the untreated condition.

### ROS Production

Intracellular production of reactive oxygen species (ROS) was quantified using the H_2_DCFDA fluorometric method: briefly, peritoneal macrophages were labeled with H_2_DCF-DA (20 μM; BioChemika Fluka) for 30 min and then washed with PBS before 2 × 10^5^ cells per well were seeded into black 96-well plates. Cells were incubated with C5a (50 ng/ml) or without, for 5 h before fluorescence was measured at 10-min intervals for 100 min using the Infinite M200 plate reader (Tecan; excitation 485 nm, emission 538 nm). ROS levels are displayed as the percentage increase in ROS relative to untreated controls.

### Statistical Analysis

Statistical significance was assessed using unpaired two-tailed *t* tests. Data were analyzed using Prism 7 (GraphPad).

## Results

### C5a, But Not C3a, Boosts IL-1α/β Release from Peritoneal Macrophages

**FIGURE 1:**
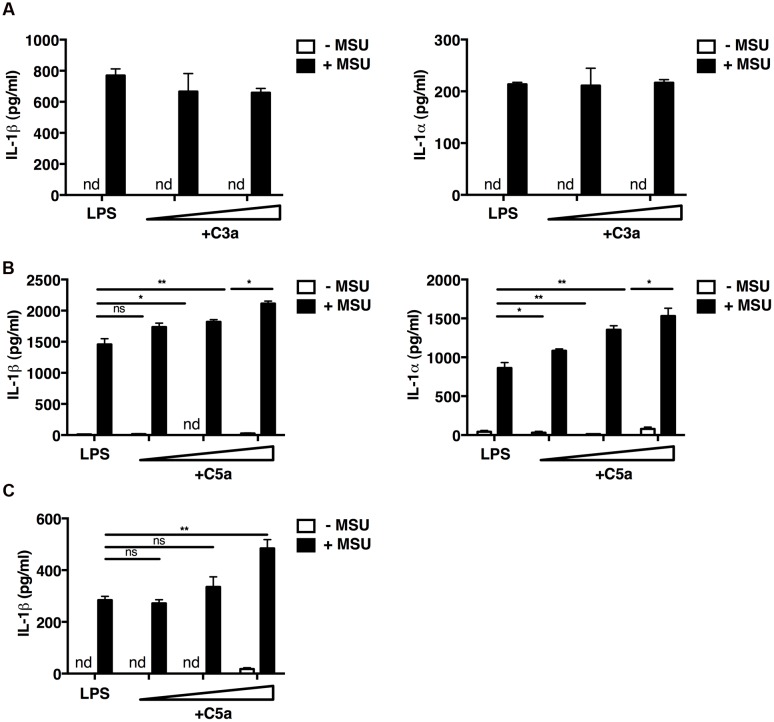
**C5a enhances IL-1α and IL-1β release from peritoneal macrophages and a human monocytic cell line.** LPS-primed mouse peritoneal macrophages **(A,B)** were incubated or not with C3a alone (0.25 and 1.25 μg/ml; **A**), C5a alone (1–10–50 ng/ml; **B**), or with C3a or C5a in combination with MSU crystals (250 μg/ml). **(C)** LPS-primed human THP1 monocytic cells were left untreated or treated with C5a alone (1–10–50 ng/ml) or in the presence of MSU crystals (250 μg/ml). Culture supernatants were collected after 24 h and IL-1α/β levels were measured by ELISA. Values are shown as means ± standard error; *n* = 3. ^∗∗^*p* < 0.01; ^∗^*p* < 0.05. nd, none detected.

We first examined the interaction of the complement components C3a and C5a with macrophages *in vitro*. Mouse peritoneal macrophages were primed with LPS, and incubated with MSU crystals or not in the presence or absence of increasing concentrations of recombinant C3a or C5a. In the absence of MSU crystals, LPS alone, LPS and C3a, LPS and C5a did not induce the release of IL-1β and IL-1α (**Figures 1A,B**); while the addition of MSU crystals triggered the release of both IL-1α and IL-1β from macrophages (**Figures 1A,B**). This mechanism is consistent with the established dual activation model of the NLRP3 inflammasome, in that LPS is required to drive expression of the IL-1β precursor and NLRP3, while a second signal, such as MSU crystals, controls inflammasome formation and activation of caspase-1.

Macrophages co-stimulated with LPS/MSU crystals and increasing concentrations of C5a boosted IL-1β and IL-1α production in a dose-dependent manner (**Figure 1B**), whereas C3a had no such effect (**Figure 1A**). Similar results were obtained when the human monocytic THP1 cell line was used (**Figure 1C**). These data indicate that IL-1α/β release from peritoneal macrophages and human monocytic cells requires both LPS and MSU crystals, and that C5a, but not C3a, boosts this inflammasome-mediated release.

### MSU-Induced Complement Activation Elicits Neutrophil and Monocyte Recruitment in a Mouse Model of Gout

**FIGURE 2:**
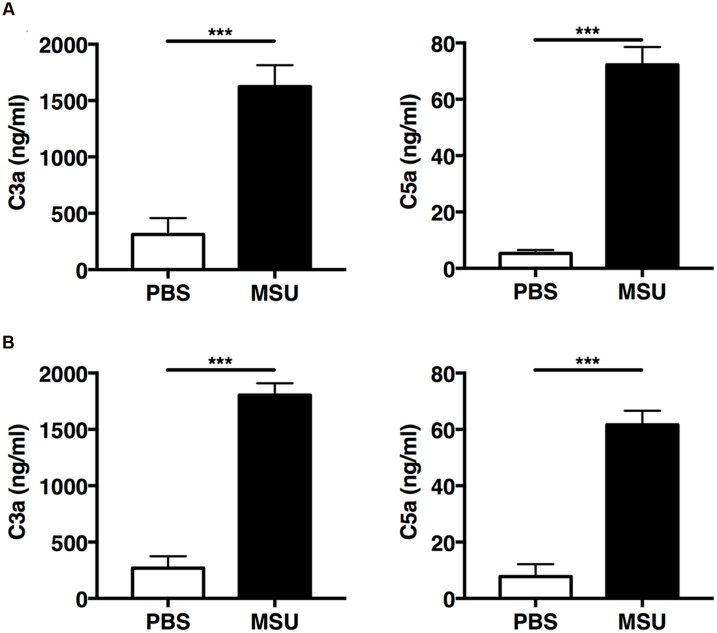
**Monosodium urate (MSU) crystals induce rapid activation of complement *in vivo*.** C57BL6 (*n* = 7; **A**) and Balb/c mice (*n* = 8; **B**) were injected with 3 mg of MSU crystals or PBS and peritoneal lavage was performed 2 h later. C3a and C5a levels in the peritoneal lavage were measured by ELISA. Values are shown as means ± standard error. ^∗∗∗^*p* < 0.001.

The mechanisms underlying complement-mediated recruitment of inflammatory leukocytes by MSU crystals *in vivo* are largely unknown. Therefore we first evaluated the ability of MSU crystals to activate the complement cascade *in vivo* by measuring amounts of C3a and C5a in the peritoneal fluid of mice following injection of MSU crystals, or vehicle only, into the peritoneal cavity of two commonly used mouse strains, C57Bl/6 and Balb/c. Two hours after MSU crystal injection C3a and C5a levels significantly increased in the lavage fluids from both C57Bl/6 (**Figure 2A**) and Balb/c mice (**Figure 2B**), relative to control animals, confirming that MSU crystals have the capacity to activate the complement cascade *in vivo*.

**FIGURE 3:**
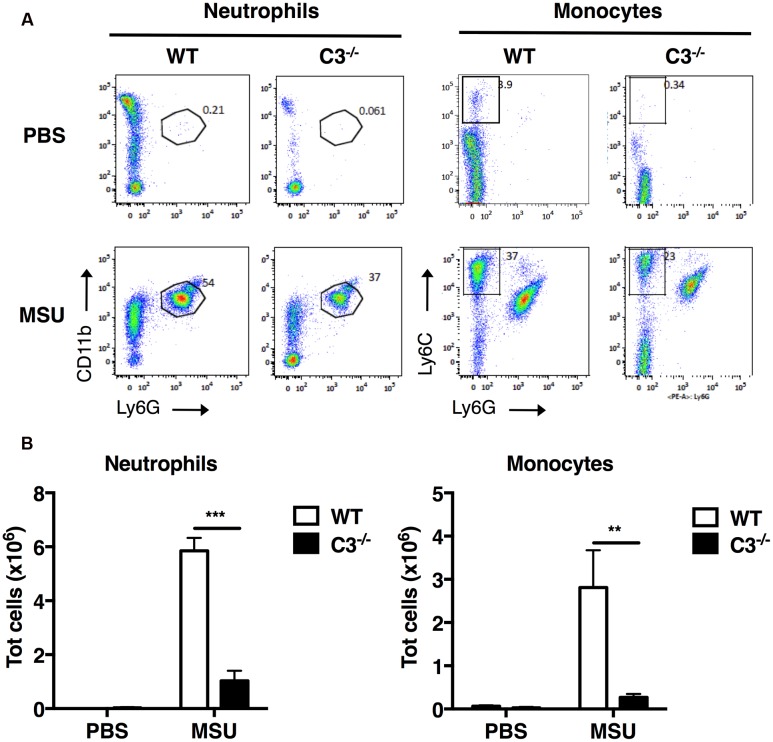
**C3 regulates recruitment of neutrophils and monocytes *in vivo* upon MSU challenge.** MSU crystals (3 mg) were injected i.p. into WT or C3^-/-^ mice. After 6 h, peritoneal cells were collected by lavage, and the numbers of neutrophils (CD11b^+^Ly6G^+^) and monocytes (CD11b^+^Ly6C^+^Ly6G^-^) counted by flow cytometry. Representative dot plots are shown in **(A)**, the mean total number of cells for each condition is shown in **(B)** as means ± standard error (*n* = 7 per condition). ^∗∗∗^*p* < 0.001; ^∗∗^*p* < 0.01.

To evaluate the importance of the complement cascade in sterile inflammation induced by MSU crystals *in vivo*, we next assessed the infiltration of neutrophils and inflammatory monocytes into the peritoneal cavity of C3^-/-^ mice, which are unable to activate the complement cascade beyond the formation of the C3-convertase. C3^-/-^ and wild-type (WT) control mice were injected i.p. with MSU crystals or vehicle; after 6 h, cells were collected by peritoneal lavage, labeled for surface molecule expression and characterized by flow cytometry. WT animals receiving MSU crystals exhibited a robust inflammatory response, characterized by massive peritoneal infiltration of neutrophils and monocytes, compared to mice receiving vehicle (**Figures 3A,B**). MSU-elicited mobilization of neutrophils and monocytes in the peritoneal cavity of C3^-/-^ mice was significantly suppressed (**Figures 3A,B**), indicating that complement proteins play an important role in regulating neutrophil and monocyte recruitment in mice, thereby promoting gouty inflammation.

### C5a is the Major Contributor to Leukocyte Infiltration during MSU-Induced Peritonitis

**FIGURE 4:**
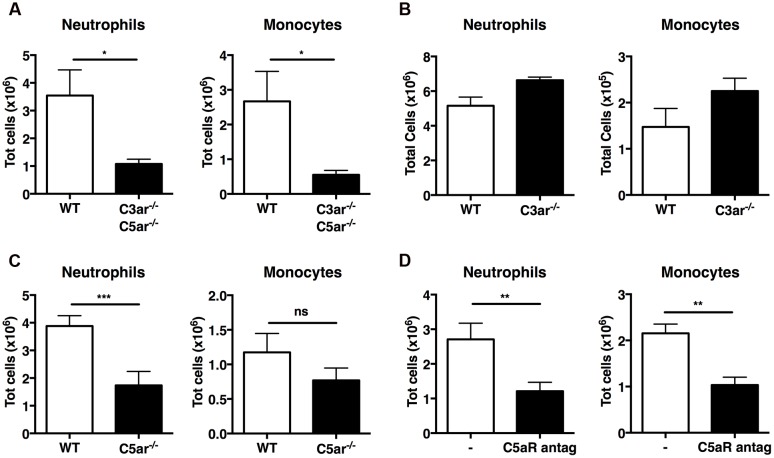
**C5a, but not C3a, regulates recruitment of neutrophils *in vivo* upon MSU challenge.** Neutrophil and monocyte recruitment was measured in the peritoneal cavity of WT (*n* = 6), C3ar1^-/-^C5ar1^-/-^ (*n* = 4; **A**), C3ar1^-/-^ (*n* = 4; **B**) and C5ar1^-/-^ (*n* = 6; **C**) mice 6 h after MSU crystal injection. **(D)** Mice (*n* = 6) received an i.p. injection of a linear C5aR antagonistic peptide prior to receiving MSU crystals. Leukocyte influx was then assessed after 6 h by flow cytometry. ^∗∗∗^*p* < 0.001; ^∗∗^*p* < 0.01; ^∗^*p* < 0.05. nd, none detected.

Active C3a and C5a are potent inflammatory mediators and chemoattractants for phagocytic cells, including neutrophils and monocytes. Early case reports found increased levels of C3a/C3adesArg in two patients with gout (Moxley and Ruddy, 1985; Jose et al., 1990); we therefore investigated whether C3a and C5a generated by MSU crystals *in vivo* are responsible for leukocyte infiltration. First, we induced MSU-elicited peritonitis in mice deficient in both C3a and C5a receptors (C3ar1^-/-^C5ar1^-/-^): significantly fewer neutrophils and monocytes were present in peritoneal lavage fluid from C3ar1^-/-^C5ar1^-/-^ mice compared with WT controls (**Figure 4A**), indicating that anaphylatoxin-mediated signaling regulates leukocyte infiltration following MSU administration *in vivo*.

To refine our understanding of the roles played by C3a and C5a in MSU-elicited peritoneal neutrophil recruitment *in vivo*, mice deficient in C3aR alone, C5aR alone, and WT controls were injected i.p. with MSU crystals. Significantly fewer neutrophils were present in the peritoneal lavage fluid of C5ar1^-/-^ mice (**Figure 4C**), but not C3ar1^-/-^ mice (**Figure 4B**), compared to WT controls, indicating that C5aR, but not C3aR, signaling is crucial for neutrophil infiltration. We also observed a tendency toward a reduction of monocyte recruitment in C5ar^-/-^ mice compered to WT controls, but this difference does not reach statistical significance.

Human C5a receptor cyclic/linear antagonistic peptides have been used to suppress inflammation in experimental rodent models of inflammatory bowel disease, ischemia/reperfusion injury, and immune complex glomerulonephritis (Paczkowski et al., 1999; Short et al., 1999; March et al., 2004; Alexander et al., 2015). Therefore, we sought to determine whether a C5aR antagonist could also suppress C5a-mediated recruitment of neutrophils and monocytes in mice after MSU challenge. WT mice were treated i.p. with the C5aR antagonist or vehicle prior to MSU challenge; peritoneal lavage after 6 h revealed that mice pretreated with the C5aR antagonist peptide had significantly fewer neutrophils and monocytes in their peritoneal cavity compared to mice receiving vehicle alone (**Figure 4D**). Our results support a central role for C5a-mediated signaling in the MSU-elicited pathology caused largely by neutrophil infiltration in mice.

### C5a Regulates IL-1β Production *In vivo* Induced by MSU Crystals

The importance of neutrophils and proinflammatory cytokines in MSU-induced inflammation led us to analyze, in more detail, the mechanisms underlying neutrophil recruitment induced by C5a. Recently, basic and clinical research studies have implicated IL-1β and its maturation by the NLRP3 inflammasome in the pathogenesis of gout (Martinon et al., 2006; So et al., 2007; Kono et al., 2010). IL-1β released from macrophages activates IL-1 receptors on epithelial cells and resident macrophages, leading to the release of pro-inflammatory cytokines and chemokines, which, in turn, recruit and activate leukocytes, amplifying the inflammatory positive-feedback loop (Kono et al., 2010). This inflammatory cascade is the major cause of gout. IL-1 receptor antagonists, such as IL-1RA or Anakinra, or anti-human IL-1β antibodies, suppress the acute neutrophil response to MSU challenge in mice (So et al., 2007; Schlesinger, 2012; Ottaviani et al., 2013; Goh et al., 2014), providing robust evidence that this pathway is major target for beneficial therapeutic interventions.

**FIGURE 5:**
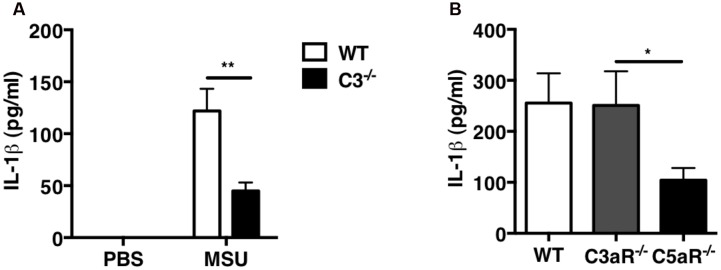
**IL-1β production is impaired in C3^-/-^ and C5ar^-/-^ mice after MSU challenge *in vivo*.** IL-1β levels were measured in peritoneal lavage fluids from WT (*n* = 6), C3^-/-^ (*n* = 6; **A**), C3ar1^-/-^ (C3aR^-/-^; *n* = 5), and C5ar1^-/-^ (C5aR^-/-^; *n* = 13) mice **(B)** 2 h after i.p. injection of MSU crystals. Values are shown as means ± standard error. ^∗∗^*p* < 0.01; ^∗^*p* < 0.05.

Because we previously demonstrated that C5a is crucial for leukocyte recruitment upon MSU challenge *in vivo*, we next sought to determine whether C5a regulates IL-1β production. IL-1β levels were measured in the peritoneal lavage fluid collected from WT and C3^-/-^ mice: IL-1β levels were significantly lower in C3^-/-^ mice compared with WT mice (**Figure 5A**). To assess the contribution of C3a and C5a signaling in IL-1β production induced by MSU crystals *in vivo*, IL-1β levels were also measured in peritoneal lavage of C3ar1^-/-^ (C3aR^-/-^) or C5ar1^-/-^ (C5aR^-/-^) mice: MSU administration induced abundant and comparable IL-1β production in WT and C3ar1^-/-^ mice, but far less so in C5ar1^-/-^ mice (**Figure 5B**). These results indicate that C5a generated by complement activation induced by MSU triggers IL-1β production *in vivo*, which in turn regulates neutrophil recruitment.

### C5a Triggers ROS Production in Macrophages

**FIGURE 6:**
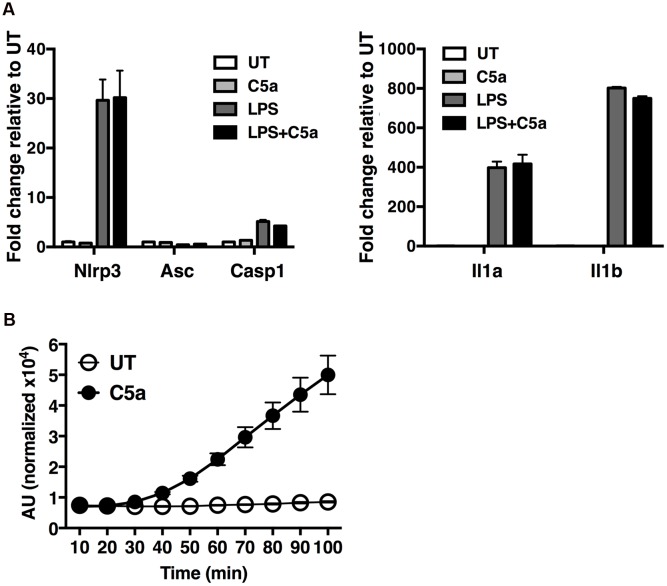
**C5a robustly elicits ROS production in peritoneal macrophages. (A)** Expression of Nlrp3, Asc, Casp1, Il1b and Il1a genes was measured in macrophages stimulated with LPS alone (0.1 μg/ml), C5a alone (50 ng/ml) or LPS in combination with C5a, by real-time quantitative RT-PCR. Results were calculated using the ΔΔCt method and data presented as mean +/- standard deviations of three biological replicates. **(B)** ROS production evaluated as H_2_DCF-DA incorporation into macrophages stimulated with C5a (50 ng/ml) or medium. ROS levels are represented as the percentage increase in treated samples relative to untreated samples (UT) and the error is represented as the standard deviation. AU, arbitrary units.

To gain insight into the molecular mechanisms by which C5a induces IL-1β in peritoneal macrophages, we first examined the possibility that C5aR signaling primes and/or activates the NLRP3 inflammasome. Peritoneal macrophages were exposed to LPS alone (a classical NLRP3 priming signal), C5a alone, or LPS in combination with C5a, for 4 h before quantification of the abundance of mRNA transcripts of the inflammasome components Nlrp3, Asc and Casp1, as well as Il1b and Il1a. While LPS robustly triggered Nlrp3, Il1b and Il1a transcription, C5a alone failed to do so (**Figure 6A**), and can therefore not be considered a priming stimulus.

Next we sought to investigate alternate mechanisms by which C5a boosts NLRP3 inflammasome activation. Since ROS have been shown to act as a primary inducer of NLRP3 inflammasome activity, we asked whether C5a, alike to MSU, triggers ROS production. We found that C5a exposure robustly increased ROS production in peritoneal macrophages (**Figure 6B**), suggesting that C5a is likely to increase NLRP3 activation at least in part via ROS production.

## Discussion

**FIGURE 7:**
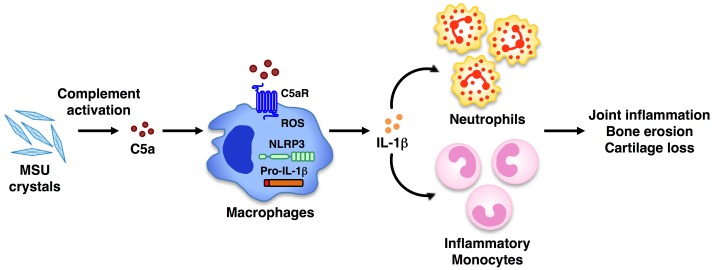
**Model of C5a-induced IL-1β release and leukocyte infiltration upon MSU crystal challenge *in vivo*.** Peritoneal administration of MSU crystals causes activation of the complement system, which leads to the generation of the anaphylatoxin C5a. Upon engagement of its own receptor, C5a in turn elicits the activation of the NLRP3 inflammasome, leading to IL-1β release. This triggers the release of chemokines from resident cells, which recruit inflammatory cells (i.e., neutrophils, monocytes) to the tissue site. Activated inflammatory leukocytes, cytokines, and chemokines contribute to the chronic inflammation that can lead to bone erosion and cartilage loss. Therapeutic blockade of C5aR signaling in humans may be an alternative new approach to ameliorate the inflammatory attack in gouty arthritis.

The anaphylatoxin C5a is generated upon complement activation and contributes to the development of many inflammatory disorders, including glomerulonephritis (Welch, 2002), pulmonary hypersensitivity (Shushakova et al., 2002), and contact hypersensitivity (Tsuji et al., 2000). C5a is a potent mediator of the inflammatory response as a result of its ability, upon engagement of C5a receptors, to regulate vasodilation and to mediate chemotaxis and activation of inflammatory leukocytes (granulocytes, mast cells, and macrophages; Merle et al., 2015b). However, whether C5a is crucial *in vivo* for gouty inflammation was unclear. In the present study, we identify C5a generated by MSU crystals *in vivo* as one of the key regulators of IL-1β release, which in turn triggers the recruitment of neutrophils, a hallmark of gouty inflammation (**Figure 7**). Specifically, C5aR deficiency was associated with less neutrophil infiltration induced by MSU crystals *in vivo*, and lower IL-1β levels.

Early studies suggested that MSU crystals can promote inflammation through complement activation: complement proteins can deposit on the MSU crystal surface leading to the activation of the classical and alternative complement pathways (Russell et al., 1982; Doherty et al., 1983). Rabbits deficient in the complement component C6, critical for MAC formation, have diminished joint swelling in a model of experimental gout arthritis, associated with decreased recruitment of inflammatory leukocytes, particularly neutrophils, and lower IL-8 levels in the joint (Tramontini et al., 2004). Our data demonstrate that C5a-mediated signaling is also crucial for the ingress of neutrophils into the peritoneal cavity in response to MSU crystals *in vivo*. Since C5ar^-/-^ mice are able to assemble MAC, our results indicate that C5a is likely to play a role that supersedes that of the MAC in sterile crystal-induced inflammation in mice.

What are the mechanisms triggering C5a-mediated IL-1 release in response to MSU crystals *in vivo*? Recent data from our and other groups have shown the critical role of the complement system in promoting NLRP3 inflammasome activation leading to IL-1β release (Laudisi et al., 2013; Triantafilou et al., 2013; An et al., 2014; Cumpelik et al., 2015). It was reported that C5a stimulation induced pro-IL-1β expression and IL-1β release in human primary monocytes in a dose-dependent manner following a mechanism that requires Ca^2+^ and K^+^ fluxes, and cathepsin B activity (An et al., 2014). C5aR blocking using an anti-C5aR antibody abolished the expression of pro-IL-1β and caspase-1 activation *in vitro* (An et al., 2014). We found that *in vitro* C5a exposure of mouse peritoneal macrophages did not trigger increased transcription of the inflammasome proteins Nlrp3, Asc and Casp1, or of Il1β and Il1α, but instead boosted ROS production. These findings highlight the fact that although the mechanism may diverge in different cell types (An et al., 2014; Cumpelik et al., 2015), C5a/C5aR interactions do provoke inflammasome activation and thereby IL-1 release. Beside IL-1, other mechanisms elicited by MSU-induced C5a *in vivo* may exist. C5a shows chemoattractant properties for neutrophils and monocytes in other model of inflammation. Thus, on one side, MSU-elicited C5a can induce production of IL-1 and on the other side, lead to chemotaxis of inflammatory leukocytes.

We have shown that MSU crystals also elicit the generation of the anaphylatoxin, C3a. The possible contribution of C3a to the mobilization of leukocytes during sterile inflammation has not been reported: we found that C3a does not synergize with the priming signal LPS in boosting pro-inflammatory cytokine production, such as IL-1α and IL-1β, from mouse peritoneal macrophages stimulated *in vitro* with LPS/MSU crystals, and that C3aR deficiency did not diminish leukocyte influx or IL-1β levels induced by MSU crystals *in vivo*. These data indicate that C3aR signaling is dispensable for acute MSU crystal-induced inflammation and highlight the distinct roles of C3a and C5a. Although C3a and C5a receptor signaling pathways share a high degree of functional overlap (e.g., cAMP, MAPK/ERK, NF-κB pathways), our data indicate that C3a is not directly involved in NLRP3 inflammasome activation and induction of IL-1β release in MSU-induced inflammation both *in vitro* and *in vivo*. It would be of particular interest in future studies to identify C5a-specific mechanisms that regulate inflammasome activation in the context of sterile inflammation, as well as to define the pro- and anti-inflammatory effector functions elicited by C5a. A recent study has shown that neutrophil-derived microvesicles isolated from human synovial fluid within 1 day of an acute gout attack exhibit anti-inflammatory effects through inhibition of C5a signaling and NLRP3 inflammasome activation (Cumpelik et al., 2015).

Studies assessing the efficacy of antibodies or antagonistic peptides blocking C5a function in gouty arthritis are lacking. However, C5 blockers are currently in use in clinical and pre-clinical studies for a number of other diseases. Eculizumab (Soliris^®^), a humanized anti-C5 monoclonal antibody, prevents C5 from being cleaved into C5a and C5b by C5 convertase, and is currently in trials for paroxysmal nocturnal hemoglobinuria (PNH) and atypical hemolytic uremic syndrome (aHUS), with plans to extend its use to age-related macular degeneration, myasthenia gravis, optic neuritis, early septic organ dysfunction, and prevention of rejection of kidney transplants (Melis et al., 2015; Risitano, 2015; Horiuchi and Tsukamoto, 2016). In addition to C5 antibodies, peptidic and non-peptidic C5aR antagonists are in clinical trials, and some have been proven efficacious (Morgan and Harris, 2015). Anti-complement agents have received attention as a new treatment strategy for refractory inflammatory diseases: considering the close link between complement and rheumatic diseases it would also be important to test anti-complement agents in these diseases. Our findings suggest the possibility that C5 inhibitors may be an alternative approach to diminish IL-1-mediated neutrophil infiltration associated with crystal-induced acute inflammation, with the potential to lead to amelioration of acute and chronic gouty arthritis.

## Author Contributions

Study concept and design: AH, FL, and AM. Study supervision: AM. Acquisition of data: HK, AH, FL, HD, and MK. Analysis and interpretation of data: HK, AH, FL, MK, BS, and AM. Technical support: HD. Writing of the manuscript: AM. Critical revision of the manuscript: HK, AH, FL, BS, and GGT.

## Conflict of Interest Statement

The authors declare that the research was conducted in the absence of any commercial or financial relationships that could be construed as a potential conflict of interest. The reviewer RL and handling Editor declared their shared affiliation, and the handling Editor states that the process nevertheless met the standards of a fair and objective review.
